# Supplementary material for: dNAGLU Extends Life Span and Promotes Fitness and Stress Resistance in Drosophila
Source: Int J Mol Sci. 2022 Nov 20;23(22):14433. doi: 10.3390/ijms232214433 (PMC9694703; doi:10.3390/ijms232214433)
Supplement: Supplementary file 1 [file ijms-23-14433-s001.zip › ijms-1990530-supplementary.pdf]

Supplementary Data

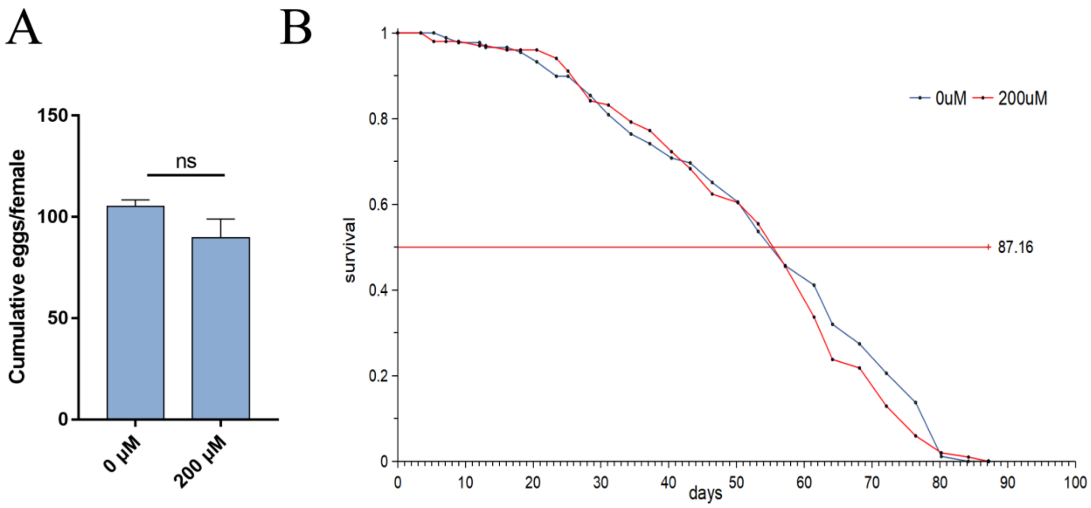

Supplementary Figure S1. Effects of 200 μM RU486 on the fecundity and lifespan in *Drosophila*: (**A**) fecundity evaluation of w^{1118} flies, $p = 0.0504$; (**B**) survival curves generated from life statistics of w^{1118} flies , $p = 0.3471$. ns represents non-significant. All survival data were analyzed with the log-rank test.

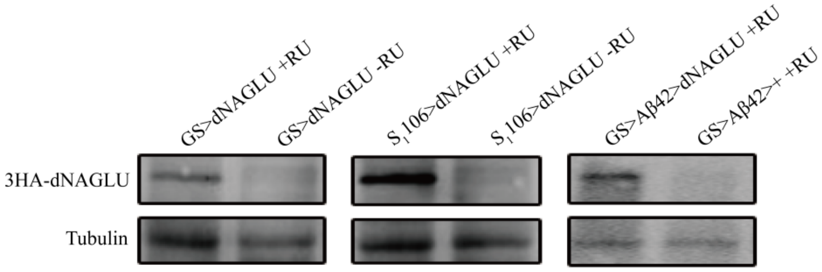

Supplementary Figure S2. Western Blot detection of 3HA-dNAGLU expression.

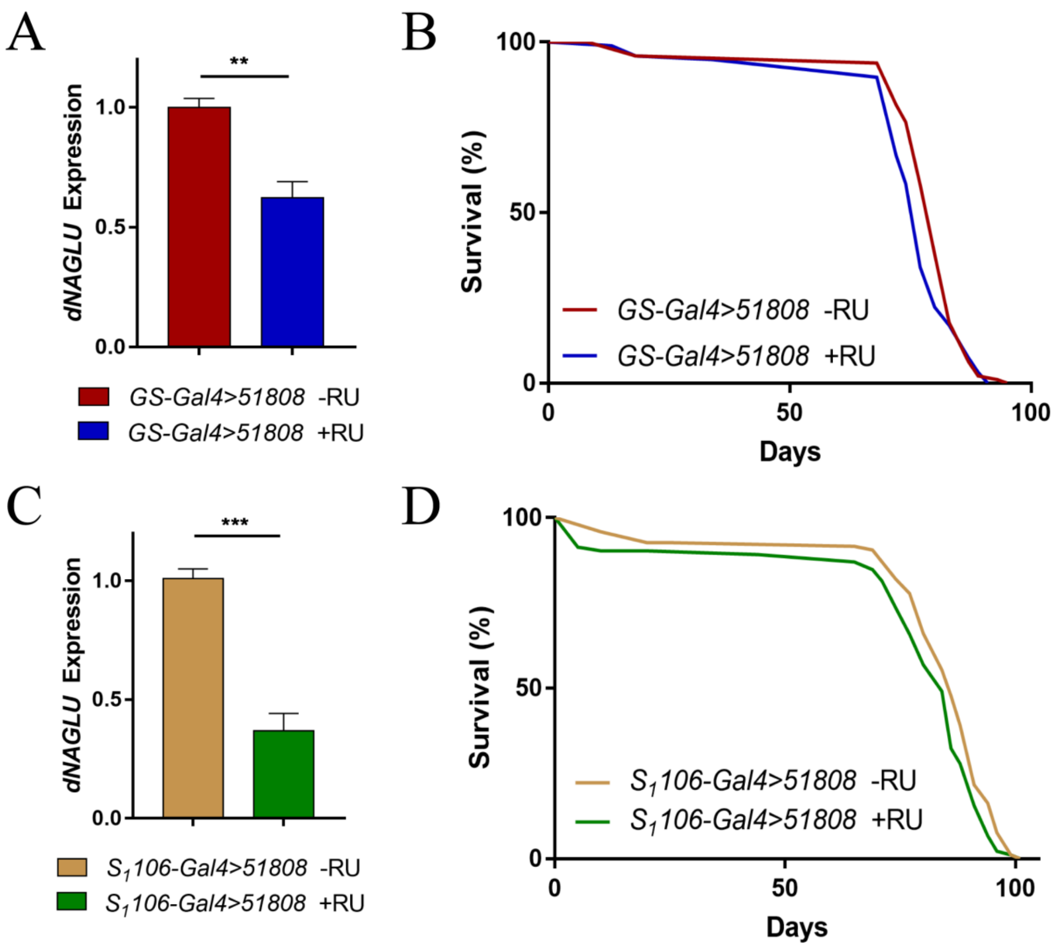

Supplementary Figure S3. Effects of *dNAGLU* knockdown on the lifespan in *Drosophila*: **(A)** qRT-PCR validation for expression of *dNAGLU* in *Drosophila* whole body; **(B)** survival curves when *dNAGLU* was knocked down in *Drosophila* whole body, p = 0.0374; **(C)** qRT-PCR validation for expression of *dNAGLU* in *Drosophila* fat body; **(D)** survival curves when *dNAGLU* was knocked down in *Drosophila* whole body, p = 0.0392. ** p<0.01, *** p<0.001. All survival data were analyzed with the log-rank test.

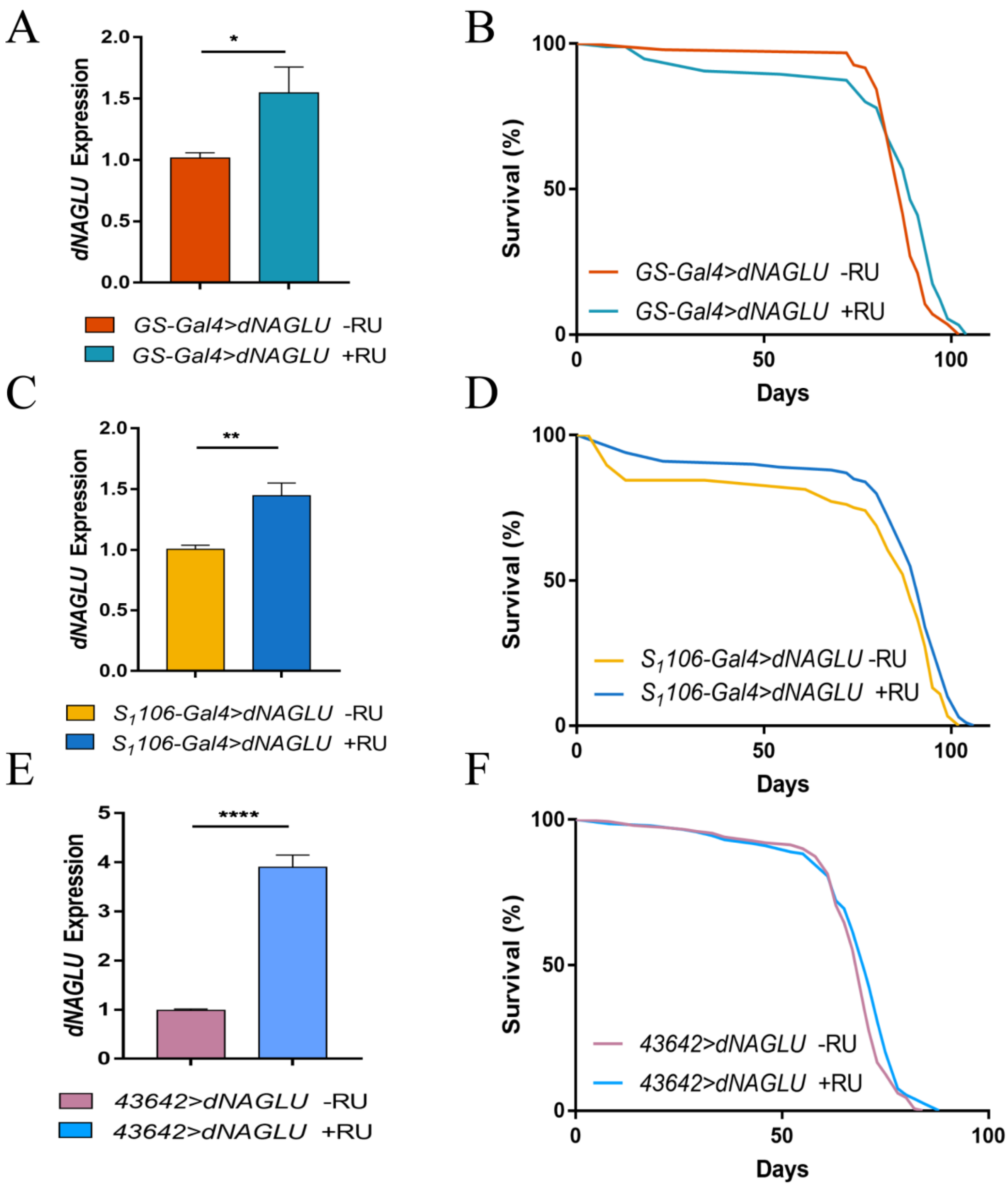

Supplementary Figure S4. Repeated experiment of *dNAGLU* overexpression on the lifespan in *Drosophila*: (A) qRT-PCR validation for expression of *dNAGLU* in *Drosophila* whole body; **(B)** survival curves of overexpressing *dNAGLU* in *Drosophila* whole body, p = 0.0235; **(C)** qRT-PCR validation for expression of *dNAGLU* in *Drosophila* fat body; **(D)** survival curves of overexpressing *dNAGLU* in *Drosophila* fat body, p = 0.0244; **(E)** qRT-PCR validation for expression of *dNAGLU* in *Drosophila* nervours system; **(F)** survival curves of overexpressing *dNAGLU* in *Drosophila* nervours system, p = 0.0259. A 5% or lower P value is considered to be statistically significant using Student's t-test, *p<0.05, ** p<0.01, **** p<0.0001. All survival data were analyzed with the log-rank test.

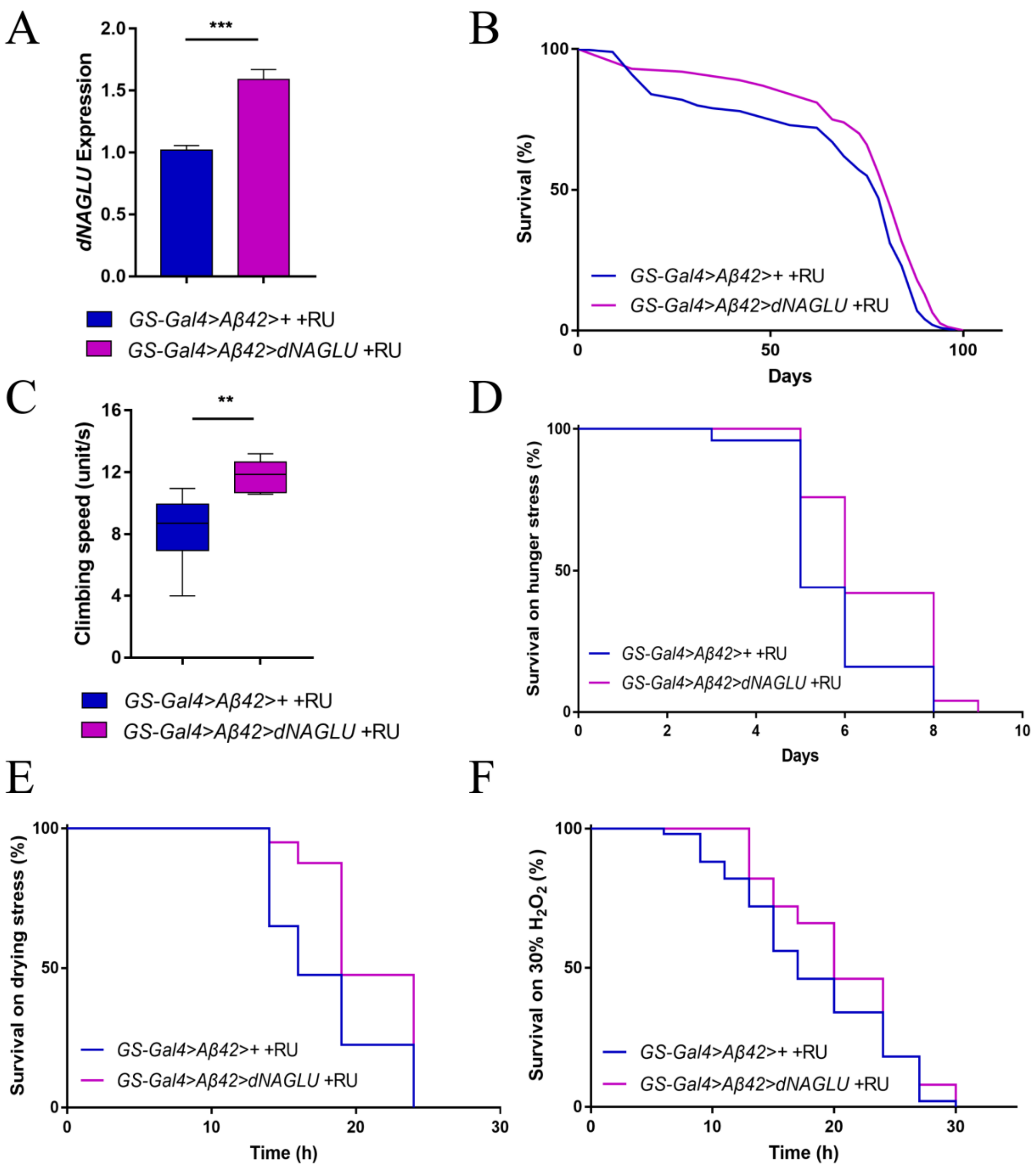

Supplementary Figure S5. Repeated experiment of *dNAGLU* overexpression on the lifespan in *Drosophila* AD models: (A) qRT-PCR validation for expression of *dNAGLU* in AD flies (*** p<0.001, t-test); (**B**) survival curve of *Drosophila* overexpressing *dNAGLU* in AD flies (p = 0.0196, log-rank test); (**C-F**) effects of *dNAGLU* overexpression on stress tolerance in AD flies; (**C**) climbing assay, (** p<0.01, t-test); (**D**) hunger stress assay, (p = 0.0003, log-rank test); (**E**) desiccation stress assay, (p = 0.0008, log-rank test); (**F**) H_2O_2 stress assay, (p = 0.1130, log-rank test).

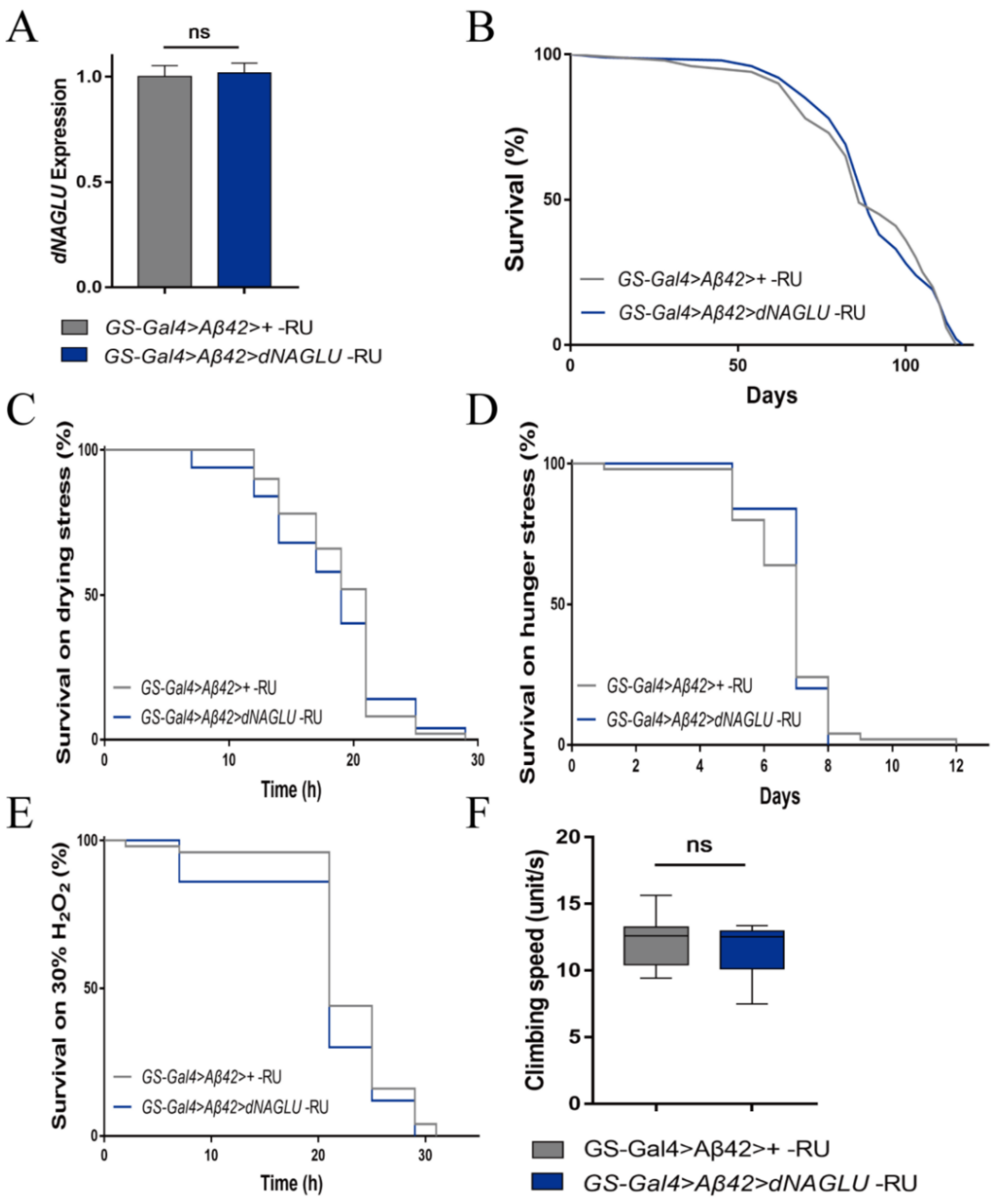

Supplementary Figure S6. There were no significant differences between the *GS>Aβ42>dNAGLU* −RU flies and *GS>Aβ42>+* −RU flies: (**A**) qRT-PCR validation for expression of *dNAGLU* ($p = 0.6929$, t-test); (**B**) survival curve ($p = 0.8473$); (**C-F**) effects on stress tolerance; (**C**) desiccation stress $p = 0.5964$; (**D**) hunger stress, $p = 0.5709$; (**E**) H_2O_2 stress, $p = 0.0872$; (**F**) climbing ability, $p = 0.5489$. ns represents non-significant. All survival data were analyzed with the log-rank test.

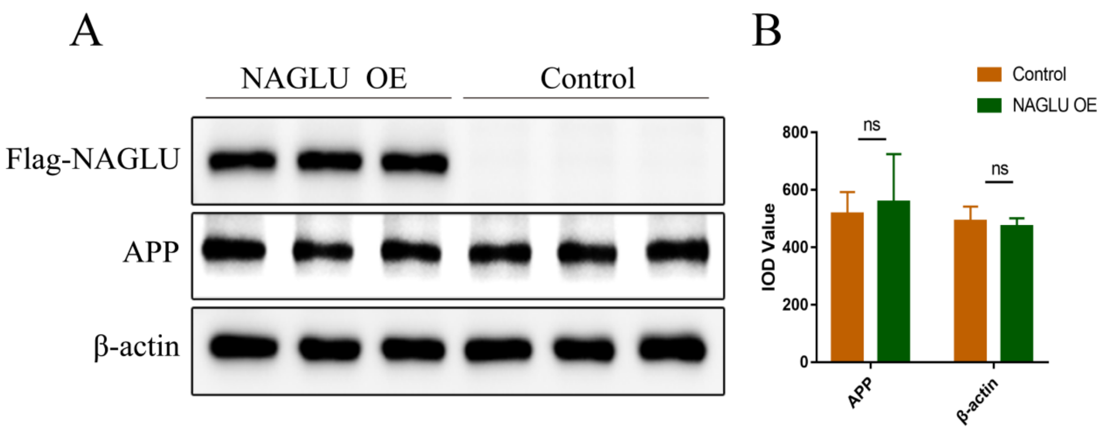

Supplementary Figure S7. Western Blot detection when *NAGLU* overexpressed in U251-APP cells: (**A**) Western Blot detection of Flag-NAGLU, APP and β-actin; (**B**) IOD value of APP and β-actin. ns represents non-significant.

Table S1. Primers used in the cloning and qRT-PCR.

Primer	Sequence
dNAGLU-F	GCTGAATTCGTTAACAGATCTATGCAGCTGAATTGGAAGCTG
dNAGLU-R	CAAAGATCCTCTAGAGGTACCTTATTTAGGTCCCGCTTTTTGAG
dNAGLU-qF	AGCTGAAAGTCTCGCACGAA
dNAGLU-qR	GCGTGTCCTTAGAGTAGCCC
NAGLU-F	GATTCTAGAGCTAGCGAATTCATGGACTACAAAGACGATGACGAC<br>AAGATGGAGGCGGTGGCGGTGG
NAGLU-R	ATCCTTCGCGGCCGCGGATCCTCACCAAGAGCCGGCCACCCAG
NAGLU-qF	CTACCTGCGCGACTTCTGTG
NAGLU-qR	GGTAGGCGTCTTCTTTCCGT
GAPDH-F	GAGCCCGCAGCCTCCCGCTT
GAPDH-R	CCCGCGGCCATCACGCCACAG
Tubulin-F	GCTTTCCCAAGAAGCTCATACA
Tubulin-R	GGTTCAGTGCGGTATTATCCAG
Actin5C-F	CGGTATCGTTCTGGACTCCG
Actin5C-R	GCGGTGGTGGTGAAAGAGTA
CTSB-F	AGAGTTATGTTTACCGAGGACCT

CTSB-R	GATGCAGATCCGGTCAGAGA
CTSD-F	TGCTCAAGAACTACATGGACGC
CTSD-R	CGAAGACGACTGTGAAGCACT
CTSK-F	ACACCCACTGGGAGCTATG
CTSK-R	GACAGGGGTACTTTGAGTCCA
CTSL-F	CTTTTGCCTGGGAATTGCCTC
CTSL-R	CATCGCCTTCCACTTGGTC
TPP1-F	CCTCCACACGGTGCAAAAATG
TPP1-R	CTCTGCTTGTCGGATGCTCAG
MMP-2-F	TACAGGATCATTGGCTACACACC
MMP-2-R	GGTCACATCGCTCCAGACT
MMP-9-F	TGTACCGCTATGGTTACACTCG
MMP-9-R	GGCAGGGACAGTTGCTTCT
ACE-F	GGAGGAATATGACCGGACATCC
ACE-R	TGGTTGGCTATTTGCATGTTCTT
TFEB-F	ACCTGTCCGAGACCTATGGG
TFEB-R	CGTCCAGACGCATAATGTTGTC
TFE3-F	CCGTGTTCGTGCTGTTGGA
TFE3-R	GCTCGTAGAAGCTGTCAGGAT
